# Tissue remodeling by invadosomes

**DOI:** 10.12703/r/10-39

**Published:** 2021-04-16

**Authors:** Alessandra Cambi, Philippe Chavrier

**Affiliations:** 1 Department of Cell Biology, Radboud Institute for Molecular Life Sciences, Radboud University Medical Center, Nijmegen, The Netherlands; 2 Institut Curie, PSL Research University, CNRS UMR144, Paris, France

**Keywords:** Invadosome, invadopodium, podosome, actin cytoskeleton, cell migration

## Abstract

One of the strategies used by cells to degrade and remodel the extracellular matrix (ECM) is based on invadosomes, actin-based force-producing cell–ECM contacts that function in adhesion and migration and are characterized by their capacity to mediate pericellular proteolysis of ECM components. Invadosomes found in normal cells are called podosomes, whereas invadosomes of invading cancer cells are named invadopodia. Despite their broad involvement in cell migration and in protease-dependent ECM remodeling and their detection in living organisms and in fresh tumor tissue specimens, the specific composition and dynamic behavior of podosomes and invadopodia and their functional relevance *in vivo* remain poorly understood. Here, we discuss recent findings that underline commonalities and peculiarities of podosome and invadopodia in terms of organization and function and propose an updated definition of these cellular protrusions, which are increasingly relevant in patho-physiological tissue remodeling.

## Introduction

Tissue remodeling is the (patho)physiological reorganization or renewal of existing tissues and consists of changes in extracellular matrix (ECM) composition and architecture and ensuing cellular responses. Many cells degrade and remodel the ECM using invadosomes, which are actin-based force-producing cell–ECM contacts functioning in adhesion and migration^1^. Characterized by their capacity to mediate pericellular proteolysis of ECM components, the invadosomes formed by non-transformed cells are called podosomes^2,3^, whereas invadosomes of invading cancer cells are named invadopodia^4^. These structures are broadly involved in cell migration and in the protease-dependent invasion program of tumor cells and have been detected in living organisms and in fresh tumor tissue specimens^5–7^. Tumor molecular subtypes and the microenvironment await more systematic analyses to determine whether the capacity to form ECM-degradative invadopodia is a generic trait of all invasive metastatic cells and whether the intensity of the invadopodia response varies depending on the cancer type.

Podosomes and invadopodia likewise consist of a core of branched actin filaments, depending on N-WASP/WASP-mediated activation of the Arp2/3 complex, are sensitive to the substrate’s stiffness, and recruit matrix metalloproteinases (MMPs), mainly the trans-membrane membrane type I (MT1)-MMP and the secreted MMP2 and MMP9, to degrade the ECM^1,4,7–9^. On the other hand, podosomes and invadopodia differ in their lifetime (over 1 hour for invadopodia as compared with minutes for podosomes) and in the repertoire of upstream activators of N-WASP/WASP–Arp2/3 stimulation^10^, which also suggests differences in regulation, molecular composition, and overall architecture. It should be noted, however, that podosomes of non-cancer cells can be involved in diseases. A typical example is the involvement of osteoclast podosomes in osteoporosis^11^. A detailed understanding of podosome- and invadopodia-specific mechanisms driving organization and function could therefore reveal new targets to better control unwanted invadosome-mediated pathologies.

In this review, we will discuss recent findings that underline common and specific traits/features of podosome and invadopodia organization and function, highlight the increasingly relevant role of invadosomes in tissue remodeling in many different patho-physiological processes, and propose an updated definition of these actin-rich protrusions.

## Podosomes and invadopodia: the Good and the Bad (and the Ugly)

**Figure 1.**
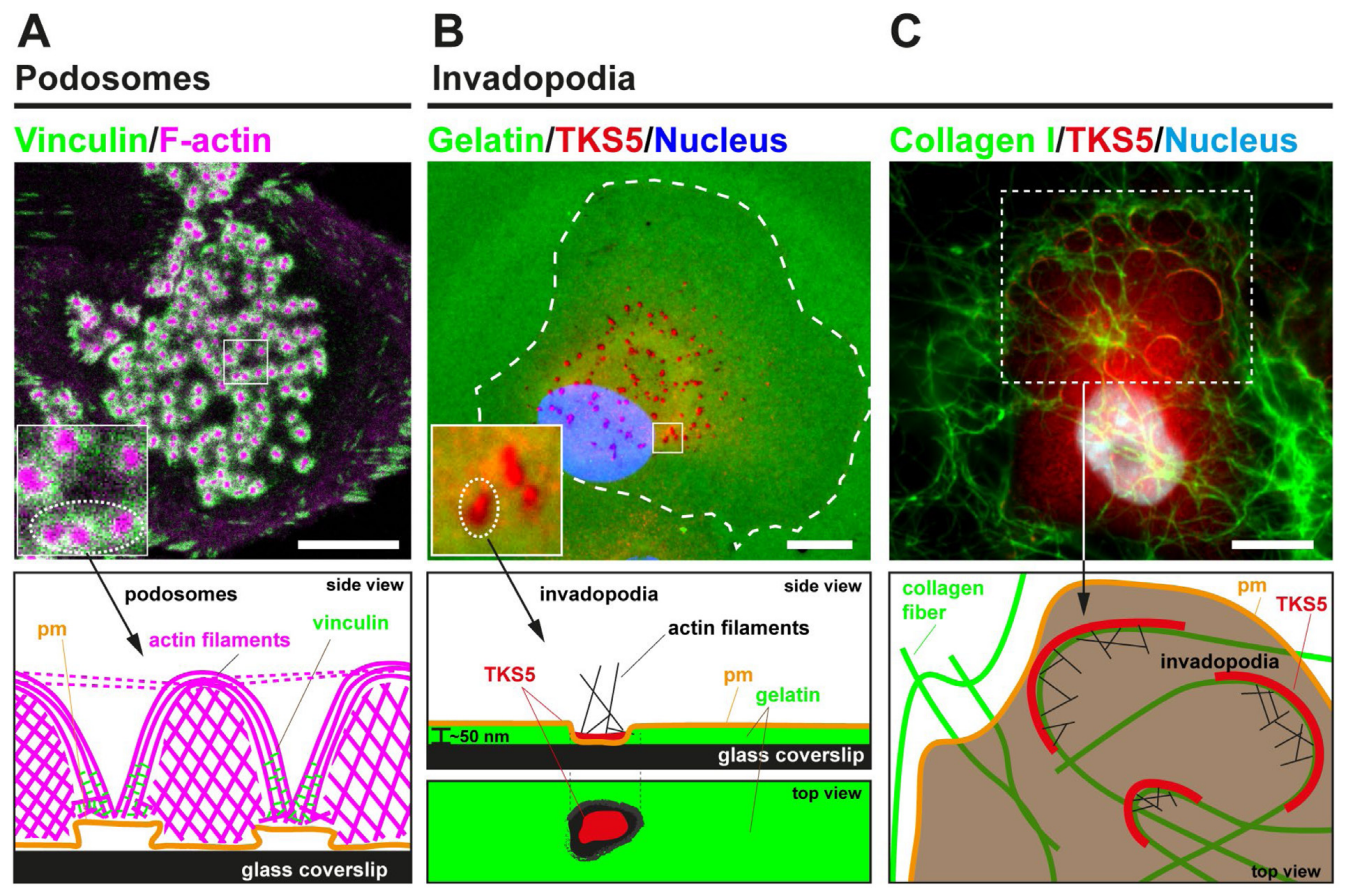
Podosome and invadopodia basics. (**A**) The upper image represents a human monocyte-derived immature dendritic cell that was plated for 60 minutes on a glass coverslip, fixed, and stained for actin (magenta) and vinculin (green). A large cluster of dot-shaped podosomes is visible at the adherent surface. The lower panel schematically depicts the side view of a few individual podosomes, highlighting the different actin architectures. (**B**) In the upper image, MDA-MB-231 cells expressing TKS5^GFP^ were plated for 60 minutes on a thin substratum of fluorescently labeled cross-linked gelatin (pseudocolored in green). Fixed cells were stained for GFP (pseudocolored in red), and the nucleus was stained with DAPI (pseudocolored in blue). Breast cancer cells form typical punctate gelatinolytic invadopodia enriched in the scaffolding protein TKS5. The lower panel schematically depicts a punctate invadopodium with TKS5 enrichment leading to the formation of a small F-actin patch lying on top of a region of gelatin degradation. (**C**) Upper image: MDA-MB-231 cells expressing TKS5^GFP^ were plated for 60 minutes on a thick layer of fluorescently labeled fibrillar type I collagen (pseudocolored in green). Cells were stained for GFP (pseudocolored in red), and the nucleus was stained with DAPI (pseudocolored in blue). The lower panel schematizes elongated TKS5-, F-actin-rich invadopodia forming at contact sites with the collagen fibers. Scale bars, 10 μm. pm, plasma membrane.

Identified back in 1985 as “cellular feet” at the adherent membrane of virus-transformed fibroblasts^12^, podosomes have been increasingly reported to form in a large variety of cell types such as leukocytes, osteoclasts, endothelial cells, and megakaryocytes^1^. In addition, ECM degradation-competent podosome-like structures have been recently identified in the neuromuscular junctions in vertebrate muscle cells^13^ and even in the parasite *Entamoeba histolytica*, which uses them to facilitate colonic tissue invasion^14^. Characterized by their round shape, individual podosomes (~0.5–1 μm Ø) consist of a central core enriched in actin and actin-binding proteins surrounded by a ring of adhesion receptors and cytoskeletal scaffolding and signaling components (Figure 1A). Depending on the cell type, multiple individual podosomes are spatially organized in large assemblies acting as mechanosensing platforms in dendritic cells, as belts in osteoclasts, and as rosettes in endothelial cells^2^.

Podosome formation and activity are associated with several pathophysiological processes in which matrix degradation plays an important role. For example, endothelial podosomes are involved in angiogenesis by breaching the basement membrane and allowing new cell–cell interactions that are key to vascular remodeling^15–17^. In the bone, osteoclast podosomes contribute to physiological bone matrix remodeling as well as osteoporosis, which is due to excessive osteoclast activity^11,18^. A podosome-based specialized structure called the sealing zone is used by osteoclasts to adhere on bone and delimit the bone-resorbing area^19^. Targeting podosome formation and spatial organization in hyperactive osteoclasts is therefore proposed as a promising strategy to limit pathological osteolysis. Indeed, targeting the formation of the sealing zone via pharmacological inhibition of Dock5, a guanine nucleotide exchange factor for the small GTPase Rac, has been shown to prevent pathological bone loss while preserving bone formation in three mouse models for the most common osteolytic diseases^11^. Moreover, the use of fullerenol nanoparticles has been reported to suppress osteoclast differentiation and inhibit the formation of the sealing zone by blocking the formation and patterning of podosomes, making these nanoparticles interesting therapeutic agents against osteoporosis^20^. Antigen-presenting cells use podosomes for migration and mechanosensing, for breaching of the basement membrane, and possibly for aiding pathogen capture^8,21,22^. Podosomes and podosome assemblies are sensitive to the topography of the extracellular environment, as they become linear-shaped when formed against fibrillar collagen^23^ and can align and almost fuse when following substrate grooves^24^. Moreover, in dendritic cells, podosome clusters act as mechanosensing platforms^22^, and nanoscale rearrangements of individual podosome actin architecture as well as different ECM-degrading capacities are observed in response to changes in substrate stiffness^25^.

Basement membranes and the bone matrix are two-dimensional (2D) surfaces in the body, which justifies the use of classical cell culture plates and 2D substratum to investigate invadosome formation and dynamics. Seemingly, invasive tumor cells are exposed to and can breach 2D ECM constructs, such as the basement membrane separating the epithelium from the stroma of any given epithelial tissue and the endothelial basement membrane during the intravasation/extravasation steps of their metastatic journey. While we increasingly understand the molecular structure of invadosomes formed on 2D surfaces, more complex is the investigation of invadosomes in a three-dimensional (3D) environment, since their characteristic architecture changes into ECM-degrading globular assemblies at the extremity of cellular protrusions, as observed in dendritic cells, macrophages, or invasive cancer cells in 3D collagen gels^26–28^. Unraveling the environment-dependent composition, dynamic properties, and function of 3D invadosomes in relation to cell migration and tissue remodeling is the current challenge.

## Podosome vs. invadopodia organization: Beauty and the Beast

Several studies explored the organization of tumor cell invadopodia in relation with two vs. three environment dimensionalities. In the classical still-useful model to study invadopodia, cancer-derived cell lines or freshly isolated tumor cells from patients are plated on a 2D substratum of fluorescently labeled gelatin (denatured type I collagen) where degradative activity is concentrated in 0.5–1 μm diameter, cortactin/F-actin-rich dot-like invadopodia^29^ (Figure 1B). Although these structures have been broadly designated as protrusive invadopodia, electron microscopy analysis has revealed that the thickness of the crosslinked gelatin coat is typically 50–100 nm^30,31^, thus leaving limited space for protrusive activity (as a reminder, the plasma membrane is typically ~10 nm thick and the lamellipodial actin meshwork is several hundred nm wide). Possibly more relevant evidence for protrusive invadopodia came from the observation of breast or colon carcinoma cells plated on a layer of Matrigel, which polymerizes to form a hydrogel of composition resembling the basement membrane, deposited on the surface of a porous polycarbonate filter^32^. However, in this experimental construct, it was arduous to distinguish mature ECM-degrading invadopodia from protruding F-actin/cortactin-based lamellipodial structures filling the empty space in the filter pores. In addition, because Matrigel lacks native covalent crosslinks, the implication of MMPs—and invadopodia—in the penetration of tumor cells through Matrigel could be questionable^33,34^.

Using a thick (4–5 μm thick), cell-derived matrix construct consisting of a densely packed fibrillar type I collagen matrix, scientists reported numerous puncta-like invadopodia at the adherent surface of MDA-MB-231 breast cancer cells^35,36^. However, when exposed to a sparser fibrous network of acid-extracted native type I collagen, cancer cells including MDA-MB-231 cells formed elongated invadopodia, up to several microns in length, in close apposition to and fitting the shape of the matrix fibers^23,37,38^ (Figure 1C). Whether these elongated proteolytically active invadopodia are formed by the coalescence of smaller individual structures assembling in different sites along the fibers is presently unknown. When visualized in cancer cells invading within a 3D fibrillary collagen gel, elongated invadopodia formed as rings (or ring segments), which circled the leading cell protrusion extending in front of the nucleus and assembled in intimate contact with constricting matrix pores^28,38–40^. Collagen cleavage, based on MT1-MMP activity, in conjunction with the production of outward forces using the energy of actin polymerization at the level of the invadopodia^38,41^, have been shown to drive invadopodia expansion in this system and, thus, to push the matrix fibers aside to facilitate nuclear and cell penetration^38^. Therefore, while, by definition, all invadopodia types degrade the matrix, their organization, activity, and dynamics differ depending on the composition, density, and mechanical properties of the matrix, at least *in vitro*^36,42,43^.

**Figure 2.**
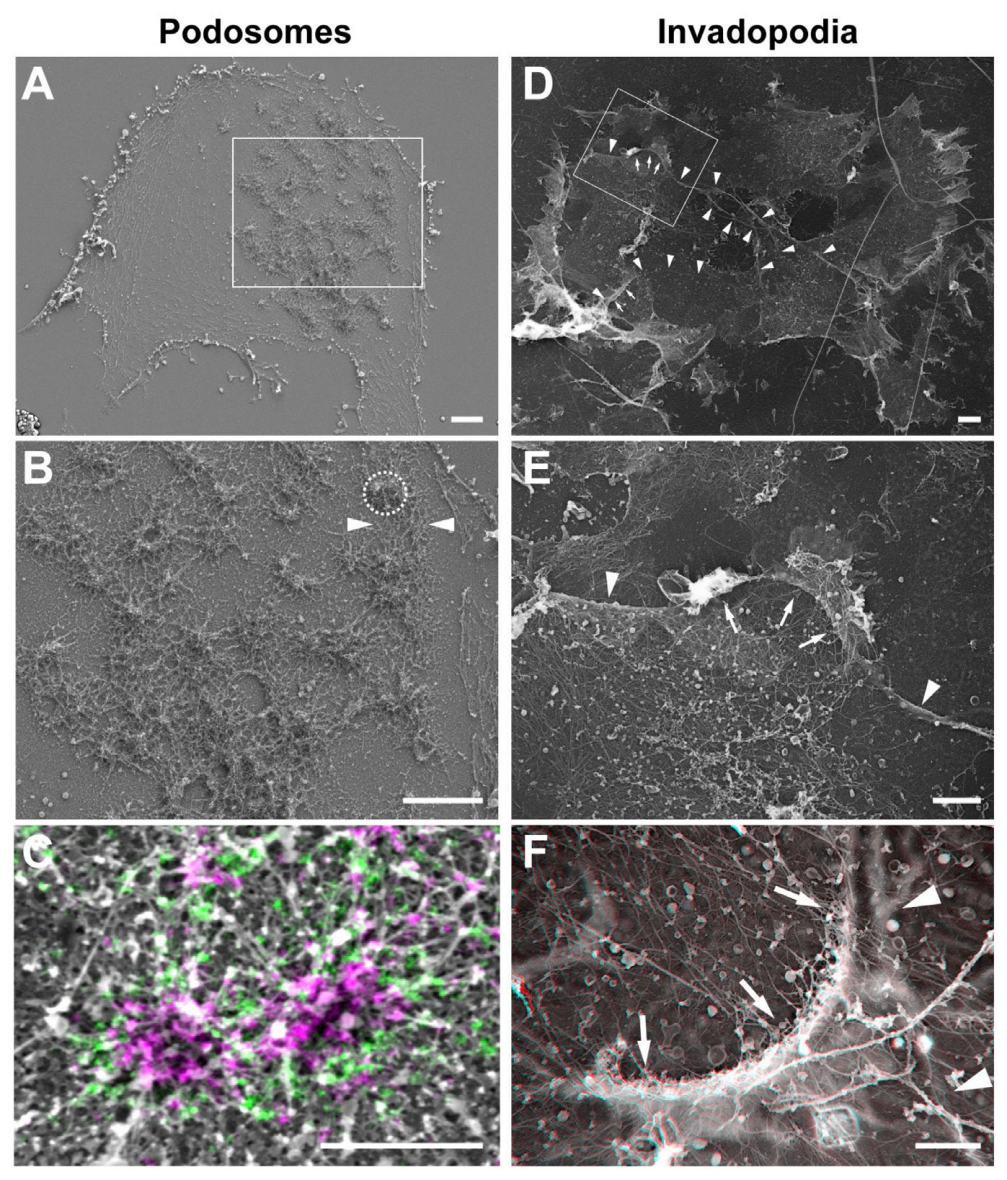
Correlative light and electron microscopy reveals the ultrastructural organization of invadopodia and podosomes. (**A**, **B**) Dendritic cells were seeded on glass coverslips, unroofed by sonication, and quickly fixed leaving ventral plasma membranes (VPMs) exposing the cytosolic side of the membrane including podosomes. After critical point drying, the VPMs were imaged by scanning electron microscopy (SEM) (gray). The core structure of a podosome is circled by a dashed line in panel **B**. Arrowheads depict inter-podosome F-actin bundles. (**C**) Fixed VPMs were stained for vinculin (green) and zyxin (purple), imaged by stochastic optical reconstruction microscopy and subsequently by SEM (gray). (**D**) Platinum replica electron microscopy survey view of the cytoplasmic surface of the adherent plasma membrane in unroofed MDA-MB-231 cells plated for 60 minutes on a thin layer of collagen I (image is inverted). (**E**) Zoomed-in region corresponding to the boxed region in panel **D**. (**F**) High-magnification view of an invadopodium in association with a curved collagen fiber. For (**F**), use view glasses for 3D viewing of anaglyphs (left eye, red). Arrows point to invadopodia appearing as bow-shaped electron-dense proteinaceous material in association with the plasma membrane above a collagen fiber. Arrowheads depict collagen fibers underneath the VPM. Scale bars: 2 μm (panels **A**, **B**, **D**, and **E**) and 1 μm (panels **C** and **F**).

A common theme to both podosomes and invadopodia is that these structures are designed to maintain intimate and prolonged contacts between surface-exposed MT1-MMP accumulated in the invadosome plasma membrane and the underlying matrix substrates to ensure optimal exploration and penetration of the pericellular tissues. Invadosomes are built upon a network of branched actin filaments depending on the activity of the Arp2/3 complex, which initiates branched actin filament growth from the sides of pre-existing mother filaments. A high degree of organization of this network is necessary to optimize the relationship between actin assembly and local production of pushing forces^44^. Along this line, several recent reports based on super-resolution microscopy highlighted a complex organization of a two-component actin network in the protrusive actin-core of individual podosomes^2,25,45^ (Figure 2A–C). As a result, these auto-assemblies function as micron-sized protrusive machineries^46–48^. In addition, interconnectivity of neighboring podosomes through a radiating network of actin filament bundles provides a higher-order organization, which ensures exploration of the pericellular matrix at a mesoscale level^22,25^. Protrusive forces in the pN range have been measured at the level of podosomes in macrophages based on the deformation of a compliant sheet of polyvinyl formal resin (see 2 and references herein). Interestingly, direct measurement of forces in the integrin receptors accumulating in the adhesion ring surrounding the podosome actin core using DNA-based FRET-FLIM probes revealed forces also in the pN range^49^. All together, these findings suggest that podosome protrusion forces may be counterbalanced by local traction forces at the podosome ring^2^.

By comparison to the podosome’s exquisite organization^50,51^, recent ultrastructure analysis by platinum replica electron microscopy revealed a rather rudimentary invadopodia architecture, consisting of a ~200–300 nm array of Arp2/3 branched actin filaments with their (+)-ends facing the plasma membrane/collagen fiber contact zone^38^ (Figure 2). Despite a simple actin meshwork organization, invadopodia can efficiently push collagen fibers away using energy from Arp2/3 complex-dependent actin polymerization^38^. Additionally, it was recently reported that seemingly rudimentary punctate invadopodia forming on the 2D gelatin matrix construct exert pN forces towards the substratum^41^. In elongated collagenolytic invadopodia, pushing forces seem to be assisted by the curvature of the actin meshwork and contacting matrix fiber, increasing frictional forces between individual actin filaments in the meshwork. Additionally, enzymatic cleavage of the matrix fibers by MT1-MMP increased matrix compliance and facilitated fiber displacement^38^. Interestingly, the collagen type I receptor discoidin domain receptor 1 (DDR1) has been reported to accumulate at the surface of cells derived from breast, lung, and liver cancer at contact sites with the surrounding collagen fibers^52,53^. Moreover, DDR1 was required for the formation of linear matrix-degradative invadopodia by these cell lines, suggesting a role for this collagen receptor in the binding and/or recognition of the collagen fibers leading to invadopodia formation^52,53^. However, contrasting with these reports, forced expression of the DDR1 receptor was found to inhibit invadopodia formation by mesenchymal (DDR1-negative) and highly invasive MDA-MB-231 breast cancer cells, while silencing of DDR1 in breast epithelial MCF10DCIS cells promoted collagenolytic invadopodia^38^. Of note, MT1-MMP, which can also interact with the collagen fibers through its extracellular hemopexin domain, was found to be required for the formation of invadopodia by breast tumor cells^38,52,53^. Therefore, more work will be required to decipher the roles played by collagen receptors including MT-MMP, DDRs, and integrins in ECM remodeling by cancer cells.

## Invadosomes in real life

Although invadopodia have been functionally linked to processes involving ECM remodeling based on numerous studies using tumor cells cultured *in vitro* and tumor cell implantation models, data documenting the existence and activity of invadopodia structures in human tumor specimens using classical, typically low-resolution imaging approaches such as immunohistochemistry on paraffin-embedded or frozen tissue sections have proven elusive. Yet the expression levels of MT1-MMP and several invadopodial components, including the key invadopodia scaffolding protein, Tks5, have been linked with poor clinical outcome in various cancers^1,54^. Along this line, in breast cancer, elevated MT1-MMP expression is required for the transition from non-disseminated *in situ* tumors to invasive carcinomatous lesions, requiring transmigration of carcinoma cells across the basement membrane, and is associated with higher metastatic risk^54–56^. In addition, genuine invadopodia structures have been observed in freshly explanted primary human tumor cells^7^. Collectively, these data imply a threatening role for invadopodia and their sword arm, MT1-MMP, during cancer dissemination and metastasis^57^. The difficulty to provide direct evidence of invadopodia occurrence in human tumors is probably related to their small size and transient nature and the lack of dedicated markers to unambiguously identify invadopodia. In this respect, the recent immunohistochemical detection of the exclusive podosome/invadopodia marker, TKS5, in patient tumor specimens represents a significant promising step^6,58^. Given the pivotal role of invadopodia in cancer cell dissemination, drugs able to counteract their function could be highly valuable for the prevention of cancer metastasis. Several reviews already detail the failure of small molecule pan-MMP inhibitors (for instance, see 59), leading to the conclusion that inhibitors with better selectivity and new strategies including targeted delivery are in need. Among the new MMP inhibitors, function-blocking antibodies directed against MT1-MMP have been developed and are characterized in preclinical studies^60,61^. Proof-of-concept unbiased screening approaches pioneered by the Courtneidge lab also successfully identified small molecules targeting invadopodia formation and function, including promising CDK5 and TAO3 kinase inhibitors^62,63^.

In the past few years, intravital microscopy of tumor cells expressing fluorescently tagged invadopodia markers offered an alternative powerful approach, and evidence for the existence of invadopodia in various genetically defined mouse cancer models has been accumulating. Intravital imaging of orthotopic mammary tumor xenografts expressing cytosolic GFP or GFP-labeled cytoskeleton and invadopodial components, cortactin, or coronin-1C revealed mostly protrusive globular structures forming at the invasive front of the cells, some of them in close proximity with interstitial type I collagen bundles detected by second harmonic generation^64–68^. In some of these studies, injection of an activatable fluorescent MMP biosensor (MMPSense) in the mouse vasculature revealed an association of cortactin-enriched protrusions with MMP activity and ascertain that these structures were proteolytically active invadopodia associated with tumor-disseminating cells^65^. In addition to providing compelling evidence that invadopodia contribute to the stromal dissemination of tumor cells, high-resolution intravital imaging using a chick embryo chorioallantoic membrane model also demonstrated that cancer cells exploit invadopodia to breach the endothelium during extravasation^69^. Very recently, tumor cell dissemination has been imaged, with actin- and cortactin-rich invasive protrusions found to be associated with degradation of the ECM and the visceral muscle layer in the native context of the *Drosophila* midgut^70^, once again highlighting the importance of simple model organisms to investigate cancer invasion.

Compared to invadopodia, podosomes *in vivo* are less well documented and imaging of their formation and dynamics in living mice by intravital microscopy is still lacking. However, several pioneering studies have provided convincing evidence of podosome formation *in vivo*. For example, the development of an *ex vivo* endothelium observation model enabled visualization of podosome rosettes and their basement membrane degradative capacity in native aortic vessel segments previously exposed to biologically active TGFβ and harvested from mice after intraventricular cardiac injection of fixative^17^. The presence of podosomes in mice aortas was also determined in vascular smooth muscle cells lacking microRNA-143/145, in which circular structures composed of cortactin and Tks5 were visualized by immunoelectron microscopy^71^. More recently, a combination of fluorescence, electron, and three‐dimensional microscopy was used to image the contacts between megakaryocytes and endothelial cells in mouse bone marrow sections, revealing that megakaryocytes use *in vivo* podosome‐like structures that collectively allow the penetration into the endothelium of bone marrow sinusoids^72^. Finally, elegant work in living *Caenorhabditis elegans* larvae showed the formation of invadosomes in the anchor cell and their role in basement membrane remodeling during vulval development^5,73^.

To better understand invadosome structure and organization in a native tissue, one would ideally want an imaging technique that allows you to obtain macroscopic views of tissues and organs and at the same time to conveniently zoom into these subcellular structures at very high resolution directly in (living) organisms. With the fast pace at which the microscopy field is moving in combination with new dedicated animal models^74^, we expect exciting discoveries in this direction in the near future.

## A revised definition of invadosomes: the Transformers of cell protrusion

The term invadosomes collectively indicates two groups of cell protrusions, podosomes and invadopodia, that share many similarities but also exhibit specific traits. Considering the most recent findings in the field, we here attempt to provide an updated set of criteria that could help define these structures. We propose minimal requirements (i.e. positivity for cortactin and F-actin) and recently identified properties (i.e. mesoscale organization for podosomes^22^ or location underneath the nucleus for invadopodia^28,31^) as new criteria that should help classify a protrusion as either a podosome or an invadopodium. Considering the plasticity of invadosome structures, which include dot-like invasive protrusions as well as plasma membrane–matrix fiber contact sites with linear features, the presence of membrane protrusions could be considered as an optional criterion to classify invadosomes. However, the detailed architecture at the individual invadosome and at the cluster level is still lacking, and properties such as dynamic behavior, mechanosensitivity, and behavior in a 3D context surely need more investigation. Moreover, invadosomes are not restricted to cell migration and matrix remodeling. In fact, cellular protrusions containing typical invadosome components have also been identified in myoblast fusion^75^ and immune synapse formation^76^ as well as phagocytosis events^77^, highlighting the great plasticity of this system. We therefore expect that novel insight into the function and architecture of invadosomes and their involvement in specific cellular processes will further sharpen the definition criteria proposed here.

The role of invadosomes in tissue remodeling is becoming increasingly clear, but open questions remain. In the osteoclasts, for example, podosomes mediate degradation of the organic part of the bone matrix, leading to the release of molecules such as growth factors and other chemical signals that can affect the activity of surrounding osteoblasts. For invadopodia, however, the impact of their ECM degradation on cells surrounding the tumor, such as stromal cells or immune cells, remains to be established. In addition, efforts should be made to link the initial mechanobiology findings on podosomes and invadopodia to real pathophysiological situations in tissues, such as understanding how invadosomes respond to local ECM stiffening as found in fibrotic tissues or at interfaces with regenerative biomaterials.
